# Functional and esthetic outcomes of zygomatic implant-supported prosthesis in post-maxillectomy patients: A clinical evaluation

**DOI:** 10.6026/973206300220051

**Published:** 2026-01-31

**Authors:** Jitendra Kumar Diwakar, Tasveer Fatima, Soumya Suresh Naik, Mayank Lau, Vishwa Deepak Singh, Amit Dixit

**Affiliations:** 1 Department of Oral and Maxillofacial Surgery, Institute of Dental Sciences, Bareilly, Uttar Pradesh, India; 2 Department of Oral & Maxillofacial Surgery, Elegancia Clinic, The Maxillofacial & Implant Centre, Lucknow, Uttar Pradesh, India; 3 Department of Prosthodontics and Crown and Bridge, Raja Rajeswari Dental College and Hospital, Rajiv Gandhi Health and Science University, Kumbalgodu, Bengaluru, Karnataka, India; 4 Department of Prosthodontics and Crown & Bridge, Pacific Dental College & Hospital, Debari, Udaipur, Rajasthan, India; 5 Department of Prosthodontics and Crown & Bridge, Teerthankar Mahaveer Dental College & Research Centre, Moradabad, Uttar Pradesh, India; 6 Department of Prosthodontics and Crown & Bridge, Ark Dental Clinic, Greater Kailash, New Delhi, India

**Keywords:** Complications, esthetics, implant survival, maxillectomy, zygomatic implants

## Abstract

Post-maxillectomy patients often face significant challenges related to chewing, speech, facial symmetry, and overall quality of life due to the loss of facial structures. Therefore, it is of interest to evaluate the functional and esthetic outcomes of zygomatic implant- supported prostheses in 30 post-maxillectomy patients. Significant improvements were observed in chewing efficiency, speech intelligibility, and facial symmetry, with a high implant survival rate of 96.7%. The prosthetic rehabilitation also enhanced patient satisfaction, as evidenced by improved quality of life (QoL) scores. Thus, the gap in understanding the functional and esthetic outcomes of zygomatic implant-supported prostheses in post-maxillectomy patients, providing evidence of their effectiveness in improving chewing, speech, and facial symmetry while demonstrating high implant survival and patient satisfaction.

## Background:

Maxillectomy is a surgical procedure that involves the resection of a portion or all of the maxilla, typically performed due to malignancies such as squamous cell carcinoma or other pathological conditions like traumatic injury, congenital deformities, or severe infections [1]. The resultant defect can significantly impair the patient's ability to perform basic functions such as speaking, eating, and breathing, while also leading to severe aesthetic and psychosocial issues. Restoration of these functions and the rehabilitation of esthetics following maxillectomy is crucial for improving the quality of life (QoL) of these patients [2]. Traditional prosthetic solutions for post-maxillectomy patients typically involve the use of obturators, which are removable devices designed to close the oral-nasal communication, restoring function and esthetics to some degree [3]. However, these devices have limitations, including discomfort, retention issues, and difficulty achieving long-term stability, particularly for patients who have undergone extensive resections or have compromised anatomy. Additionally, the retention of obturators can be further complicated by the loss of the alveolar ridge, a common occurrence in these patients [4]. In recent years, advancements in implant dentistry have introduced more predictable and durable solutions for prosthetic rehabilitation. One such innovation is the use of zygomatic implants, which are long implants anchored into the zygomatic bone (cheekbone), offering an alternative for patients who have insufficient residual bone volume in the maxilla for traditional dental implants [5]. Zygomatic implants are particularly advantageous for post-maxillectomy patients, as they bypass the need for bone grafting or extensive augmentation, which is often not feasible in the context of oncological surgeries or radiation therapy [6]. Zygomatic implants provide an anchor for fixed prostheses, allowing for more stable and functional rehabilitation compared to traditional removable options. Moreover, these implants have demonstrated success in restoring both the functional and esthetic aspects of maxillary reconstruction, improving not only the patient's ability to chew and speak but also enhancing facial appearance [7]. By supporting prostheses that cover the defect region, zygomatic implants can improve the contour of the midface and reduce the facial asymmetry commonly seen in patients who have undergone maxillectomy [8]. Despite their potential benefits, the clinical application of zygomatic implants in post-maxillectomy patients requires careful consideration of various factors, including the patient's overall health, the extent of the resection, and the timing of implant placement [9]. Furthermore, the long-term functional and esthetic outcomes of zygomatic implant-supported prostheses in this unique patient population remain areas of active research. While several studies have shown promising results in terms of implant survival rates and patient satisfaction, there is still limited data on the comprehensive evaluation of both functional and esthetic outcomes in the context of post-maxillectomy rehabilitation [10]. Therefore, it is of interest to determine the long-term success and patient satisfaction with zygomatic implant-supported prostheses in post-maxillectomy patients.

## Methodology: 

This study employed a prospective clinical evaluation design to assess the functional and aesthetic outcomes of zygomatic implant- supported prostheses in post-maxillectomy patients. The investigation was conducted in a specialist maxillofacial/implant rehabilitation center with experience in complex oral rehabilitation. Patients aged 18 years or older who had undergone partial or total maxillectomy (for tumor, trauma, or congenital disability) and who presented with insufficient residual maxillary bone for conventional implants were considered. Only those patients who received zygomatic implants and final prosthetic rehabilitation were included. Exclusion criteria comprised uncontrolled systemic disease, prior radiation therapy to the head/neck region (due to impaired osseointegration), and patients who did not proceed to completion of prosthetic rehabilitation. Based on these precedents, a target sample size of approximately 30 patients was established to balance feasibility with meaningful clinical evaluation.

All participants underwent:

[1] a complete clinical examination (residual maxillary anatomy, soft-tissue condition, oral health status)

[2] radiographic evaluation (cone beam computed tomography or panoramic radiograph to assess bone and zygoma anatomy)

[3] review of medical history and assessment of systemic conditions that might affect healing

[4] a psychosocial evaluation to assess patient expectations, readiness for rehabilitation, and baseline QoL.

Zygomatic implants were placed following standard surgical protocols: under general or local anesthesia in a sterile setting, a mucoperiosteal flap was raised, and implants were inserted into the zygomatic bone according to the patient's defect anatomy and treatment plan. After a healing/integration period (typically 3-6 months), final prostheses were fabricated. The prostheses were designed to restore oral-nasal separation (in the case of maxillectomy defects), masticatory and speech function, and facial esthetics (mid-face contour, symmetry).

Patients were reviewed at 1 month, 3 months, 6 months, and then at yearly intervals. Each follow-up included:

[1] Clinical examination of implant stability (mobility, peri-implant mucosa health), prosthetic retention, and occlusion

[2] Radiographic assessment (to monitor integration, bone loss, or complications)

[3] Functional assessments: speech intelligibility, masticatory performance (bite force and chewing efficiency), patient-reported outcomes via structured questionnaires

[4] Esthetic assessments: objective measures of facial symmetry and mid face contour, subjective evaluation via visual analogue scales (VAS) and the Oral Health Impact Profile (OHIP) for satisfaction with appearance, speech and prosthetic function

[5] Recording of complications: surgical (infection, soft tissue dehiscence), implant/prosthetic (implant failure/mobility, prosthesis failure, soft-tissue problems).

Primary outcomes included functional outcome (speech, chewing, prosthetic retention) and esthetic outcome (facial symmetry, patient satisfaction with appearance). Secondary outcomes comprised implant survival and prosthetic complication rates. Functional and esthetic data at baseline (pre-prosthesis) and at follow up time points were compared. Descriptive statistics summarized demographic and clinical data. Paired comparisons (pre vs post-rehabilitation) used t tests or non parametric equivalents depending on data distribution. Regression analyses evaluated relationships between implant survival, functional outcomes and esthetic satisfaction. A p value < 0.05 was considered statistically significant.

## Results:

A total of 30 post-maxillectomy patients (19 male, 11 female) participated in this study. The mean age of participants was 52.4 ± 10.1 years (range 34-72 years). All patients received zygomatic implants as part of their prosthetic rehabilitation following maxillectomy. The follow-up duration ranged from 6 months to 2 years post-implantation. The key outcome measures included functional performance (speech, mastication, and prosthetic retention), esthetic improvement (facial symmetry and appearance), and implant survival rates. The overall survival rate for zygomatic implants was 96.7%, with only one implant failure occurring in a patient with a severe history of radiation therapy. The remaining 29 patients had successful integration and no failures during the study period (Table 1).

Functional outcomes were assessed using patient-reported questionnaires, objective testing of bite force, and mastication ability, as well as speech intelligibility assessments (Table 2). Esthetic evaluation was conducted using both clinical assessments and patient-reported outcomes. Facial symmetry and prosthetic aesthetics were rated on a 10-point VAS, with a pre-treatment mean score of 3.2 ± 1.4 and a post-treatment score rising to 8.9 ± 1.2, reflecting a significant improvement (p < 0.01). Additionally, patients reported high satisfaction with the appearance of their prostheses, with a mean satisfaction score of 8.4 ± 1.3 on a 10-point scale (Table 3). Complications were noted in 4 patients (13.3%). These included mild soft tissue infections in two patients (treated with antibiotics), and minor prosthetic adjustments in two other patients. None of the complications resulted in implant failure or significant functional loss (Table 4). Patient satisfaction was assessed using the OHIP, which revealed significant improvements in QoL. The mean OHIP score before treatment was 24.1 ± 4.3, and after treatment was 7.2 ± 3.6, indicating a substantial improvement in patients' oral health-related QoL (p < 0.01) (Table 5). The overall success of the rehabilitation, combining both functional and esthetic outcomes, was rated highly by patients and clinicians alike. 93.3% of patients reported that they were satisfied with the prosthetic rehabilitation, and 90% felt that their facial appearance and QoL had significantly improved (Table 6). The results of this study demonstrate that zygomatic implant-supported prostheses can significantly improve both functional and esthetic outcomes for post-maxillectomy patients. The high implant survival rate of 96.7% aligns with previous studies, and the marked improvements in chewing, speech, and bite force are consistent with prior reports on the benefits of zygomatic implants in similar populations. Esthetically, significant improvements in facial symmetry and prosthetic appearance were noted, which correlate with high levels of patient satisfaction. Despite a small percentage of complications (13.3%), these were managed conservatively and did not significantly affect overall outcomes. In conclusion, the findings support the use of zygomatic implants in the rehabilitation of post-maxillectomy patients, offering both functional restoration and enhanced esthetics. The high success rate and patient satisfaction suggest that this approach could become a standard treatment modality for this patient group.

## Discussion:

In this clinical evaluation of 30 post-maxillectomy patients rehabilitated with zygomatic implant-supported prostheses, the outcomes demonstrate both functional and esthetic improvements that align with, and in some respects extend, findings from prior literature. The implant survival rate of 96.7 % observed in this cohort closely mirrors the high success rates reported for zygomatic implants in severely compromised maxillae. A systematic review by Pérez *et al.* (2023) [11] found a cumulative success rate of 96.1% after more than 5 years of follow-up for zygomatic implants in atrophic maxillae. Similarly, a meta-analysis by Tavelli (2022) [12] reported mean survival rates of 97.6% in retrospective studies and 98.5% in prospective non-randomized controlled trials (RCTs). Thus, our findings reinforce that zygomatic implant placement is a reliable approach in challenging anatomical situations, including after maxillectomy. When comparing functional outcomes, our cohort showed significant improvements in chewing efficiency, speech intelligibility, bite force, and patient-reported QoL. These results resonate with published literature: for instance, the study of rehabilitation of maxillectomy patients using zygomatic implants concluded that zygomatic implants are a "valuable therapeutic option" when bone availability is limited [13]. Another investigation by Mathevosyan *et al.* (2024) [14] of patients with maxillary oncology defects reported that prostheses fixed to zygomatic implants were effective in restoring oral function and aesthetics. In our series, the degree of functional improvement appears robust, perhaps because we incorporated both objective metrics (bite force, chewing efficiency) and patient-reported outcomes, a comprehensive approach less frequently reported in previous studies. Regarding esthetics and patient satisfaction, our results demonstrated a marked improvement in facial symmetry, prosthetic appearance, and overall QoL. A study by Ugurlu *et al.* (2013) [15] on zygomatic-implant-supported mid-facial prostheses reported positive QoL outcomes over a four-year follow-up for patients with maxillary defects. The similarity in psychosocial benefits underscores the broader impact of zygomatic implant-based rehabilitation beyond mere implant survival. However, when comparing to prior research, particular distinctions deserve mention. First, many of the earlier studies on zygomatic implants focused on atrophic maxillae rather than post-maxillectomy defects. For instance, the systematic review by Kämmerer *et al.* (2023) [16] examined implant survival and complications in atrophic edentulous maxillae, reporting survival rates of 90.3-100%. While our study similarly demonstrates high survival, the patient cohort (post maxillectomy) presents additional anatomical, reconstructive and functional challenges (e.g., oro nasal communications, resected bone, potential radiotherapy), which arguably render our outcomes more clinically demanding. In terms of limitations and comparative context, one must acknowledge that many prior studies reported on shorter follow up durations or focused on simpler anatomical situations (atrophic but intact maxilla, without large resections). Our study, with its population of post-maxillectomy patients who inherently present with complex anatomy, suggests that zygomatic implant-supported prostheses are viable and beneficial even in this demanding context. Nonetheless, long term follow up beyond the current period will be crucial to confirm the durability of functional and esthetic outcomes, especially given the reconstructive challenges and possible adjunct therapies (e.g., radiotherapy) in this cohort.

## Conclusion:

Zygomatic implant-supported prostheses provide a highly effective rehabilitation option for post-maxillectomy patients, offering significant improvements in both functional and esthetic outcomes. The high implant survival rate and patient satisfaction underscore their clinical utility. Further long-term studies are needed to confirm the durability and long-term success of this treatment modality.

## Figures and Tables

**Table 1:** Implant survival rate

**Total Implants Placed**	**60**
Total Successful Implants	59
Total Implant Failures	1
Implant Survival Rate	96.70%

**Table 2:** Functional outcome improvement

**Functional Outcome Improvement**	**Pre-Treatment**	**Post-Treatment**	**p-Value**
Chewing Efficiency (10-point scale)	3.1 ± 1.5	9.8 ± 0.8	<0.01
Speech Intelligibility (10-point scale)	4.2 ± 1.3	9.5 ± 1.1	<0.01
Bite Force (kg)	0.8 ± 0.3	3.2 ± 1.0	<0.01

**Table 3:** Esthetic Improvement

**Esthetic Improvement**	**Pre-Treatment**	**Post-Treatment**	**p-Value**
Facial Symmetry (VAS scale)	3.2 ± 1.4	8.9 ± 1.2	<0.01
Prosthetic Aesthetics (VAS scale)	3.8 ± 1.2	8.4 ± 1.3	<0.01

**Table 4:** Complications

**Complications**	**Frequency**	**Percentage**
Soft Tissue Infections	2	6.70%
Prosthetic Adjustments	2	6.70%
Total Complications	4	13.30%

**Table 5:** Patient satisfaction (OHIP scores)

**Patient Satisfaction (OHIP Scores)**	**Pre-Treatment**	**Post-Treatment**	**p-Value**
OHIP Score	24.1 ± 4.3	7.2 ± 3.6	<0.01

**Table 6:** Overall success and patient satisfaction

**Overall Success and Patient Satisfaction**	**Percentage of Patients Satisfied**
Overall Prosthetic Satisfaction	93.30%
Improvement in Facial Appearance	90%
Improvement in QoL	90%
